# Commentary: Psychological distress among healthcare students in Poland from COVID-19 to war on Ukraine: a cross-sectional exploratory study

**DOI:** 10.3389/fpubh.2025.1582593

**Published:** 2025-05-07

**Authors:** Mohamed Abouzid, Tomomi Hisato, Paulina Sytek, Shreya Nandy, Eman M. Monga, Alhassan Ali Ahmed

**Affiliations:** ^1^Department of Physical Pharmacy and Pharmacokinetics, Faculty of Pharmacy, Poznan University of Medical Sciences, Poznan, Poland; ^2^Doctoral School, Poznan University of Medical Sciences, Poznan, Poland; ^3^Faculty of Medicine, Poznan University of Medical Sciences, Poznan, Poland; ^4^Poznan District Hospital, Poznan, Poland; ^5^College of Graduate Health Studies, Master of Public Health Dental Emphasis, A.T. Still University, Mesa, AZ, United States; ^6^Faculty of Dentistry, Poznan University of Medical Sciences, Poznan, Poland; ^7^Department of Bioinformatics and Computational Biology, Poznan University of Medical Sciences, Poznan, Poland

**Keywords:** war, anxiety, depression, stress, healthcare students, mental health, psychache, COVID-19

## 1 Introduction

In our original paper, “*Psychological Distress Among Healthcare Students in Poland From COVID-19 to the War on Ukraine: A Cross-Sectional Exploratory Study* (1)”, we found that during the Ukrainian war and the COVID-19 pandemic, women report lower anxiety levels. However, post-pandemic anxiety remains high, while stress and depression levels are unchanged. We recommended that healthcare students, especially those away from their families, need mental, psychological, and social support.

We read the commentary by Guziak et al. with great interest and appreciate their insights. They identified a critical research gap in evaluating the effectiveness of mental health initiatives for medical students, especially in Poland, where data is scarce.

In this response, we aim to provide additional information about mental health care facilities at Polish universities and specific data on the mental health support available at Poznan University of Medical Sciences (PUMS).

## 2 Mental health of medical students in Poland

In 2021, Guziak and Walkiewicz reported that 15 of 19 medical universities (79% response rate) offered psychological support to their students (2). In 2024, they noted that 13 out of 22 medical universities (59% response rate) provided such services (3). However, there is limited information on psychological support at non-medical universities in Poland.

To fill this gap, we conducted online research from February 23 to 27, 2025, and identified 81 non-medical universities that offer mental health support in various Polish cities. Warsaw had the highest number (16), followed by Kraków County (10), Wrocław (9), and Poznań (8). Lublin had four such universities, whereas Łódz had three. Several cities—including Gdańsk, Bydgoszcz, Szczecin, Kielce, and Białystok—had two universities each. In contrast, many others (e.g., Toruń, Gliwice, Katowice, Zielona Góra, Czestochowa, Rzeszów, Wałbrzych, Piła, Siedlce, Słupsk, Tarnów, Biała Podlaska, Łomża, Płock, Opole, Gdynia, Jarosław, Nysa, Koszalin, and Oświecim) had one apiece (Supplementary File 1).

Most of these universities offer free counseling services, although some have a limited number of free sessions before charging a fee; even then, the cost is typically lower than standard private counseling. Communication methods range from email and telephone hotlines to online forms (e.g., Google Forms). We observed that some websites contained broken links, and some universities only mentioned mental health support under a “News” tab rather than having a clearly labeled support page.

Several universities referred students to the Polish Students' Parliament (Parlament Studentów Rzeczypospolitej Polskiej [PSRP]) “Comfort Zone” (Strefa Komfortu) initiative. This project, co-financed by the Ministry of Science and Higher Education, provided online therapy and student webinars nationwide. However, the resource was available only in Polish, which may have limited its usability for non-Polish-speaking students.

Another key feature of the project was a map displaying universities that offered their own psychological support services, complete with contact information and website links. However, fewer universities were listed on the map than we found through independent online searches. Universities could submit a form to be included on the map.

Unfortunately, the project ended, and visitors were encouraged to follow PSRP's social media channels for updates on the potential relaunch of *Strefa Komfortu* and psychological support services in 2025.

It is important to highlight that online searches have inherent limitations, such as outdated website information or the omission of services universities do not promote online. Moreover, Some institutions may indeed offer mental health services, yet these could not be identified online due to limited website visibility.

## 3 Discussion

PUMS has approximately 630 students in the English division (i.e., international) and 6,600 in the Polish division. For Polish students, crisis intervention psychologists operate an emergency helpline for urgent situations (e.g., suicidal ideation, psychosis, trauma). They also provide support for issues such as relationship crises, bereavement, depression, or substance abuse. Also, the Center for Adaptation and Psychological Support (PORT) offers up to 10 psychological consultations and therapy sessions. Students requiring longer-term care can receive it at the University's Mental Health Clinic or the Polish National Health Fund (NFZ). Hence, all services offered to the students are free and financed by PUMS or NFZ. The center has operated since November 2024 and is used by approximately 30 to 50 Polish students per month.

For international students, the Mental Wellness Center provides English-speaking specialists, an emergency helpline, psychological consultations, short- and long-term psychotherapy, group therapy, health coaching, and workshops on mental health. Also, there is mandatory tutoring, and each first- and second-year medical student attends at least one individual session with a psychologist or psychotherapist as part of the curriculum. The center recommends English-speaking psychiatrists for those needing psychiatric care and offers medical navigators to assist with navigating the Polish healthcare system, including help with documentation and institutional contacts. Most services at the Mental Wellness Center are free for international students of all faculties. Psychologists running the center have observed the growing interest of students in psychological help over the last 10 years, which reflects increasing awareness and acceptance of mental health support among students. In 2024, about 180 students took part in obligatory meetings, about 110 decided to participate in individual therapy, and about 50 in mental health workshops.

According to the POL-on register, 354 universities were operating in Poland during the 2023/24 academic year (4). A 2021 report from the Ministry of Education and Science surveyed 93 universities (including both public and select private institutions), and 90 responded. Among these, 85 (94%) acknowledged the need to provide psychological support to students and had implemented various initiatives (5). In response, the Ministry developed a “Psychological Package,” a set of recommendations encouraging universities to (i) create and publicize a structured psychological support plan, (ii) offer individual consultations with psychologists, and (iii) organize workshops and training sessions on mental health prevention.

The above national efforts, coupled with data from Guziak and Walkiewicz (2, 3) and our findings, indicate that although most Polish universities acknowledge the importance of mental health services, the actual availability and ease of accessing such support can vary widely—particularly in how services are promoted on university websites. We recommend a more systematic and bilingual (Polish and English) approach to advertising mental health resources, thereby improving visibility and accessibility for both domestic and international students. Additionally, we highlight that a uniform, centralized overview remains difficult to obtain, as underscored by the absence of a definitive resource enumerating how many universities in Poland offer mental health support.

Importantly, universities should prioritize mental health support as a continuous resource rather than focusing solely on crises such as the COVID-19 pandemic or geopolitical conflicts. Ensuring sustainable, high-quality mental health services for medical and non-medical students remains a pressing issue, warranting ongoing attention from academic institutions and policymakers alike.
