# Supplementary material for: Commentary: Psychological distress among healthcare students in Poland from COVID-19 to war on Ukraine: a cross-sectional exploratory study
Source: Front Public Health. 2025 May 7;13:1582593. doi: 10.3389/fpubh.2025.1582593 (PMC12092356; doi:10.3389/fpubh.2025.1582593)
Supplement: Supplementary file 1 [file Table_1.docx]

**List of psychological support by non-medical universities in Poland**

Warsaw

1. University of Warsaw

Psychological Counselling Centre of UW: services for students and employees of the University of Warsaw

E-mail: [cpp@adm.uw.edu.pl](mailto:cpp@adm.uw.edu.pl),

https://welcome.uw.edu.pl/during-your-stay/assistance-for-presons-with-disabilities-and-psychological-counselling/

2. Warsaw University of Technology

Office for university social responsibility: In order to provide all possible assistance during your studies at Warsaw University of Technology, we offer free psychological support to all our students.

Email: wsparcie.sp@pw.edu.pl

<https://bisou.pw.edu.pl/Sekcja-Psychologow>

3. Warsaw School of Economics

Psychological assistance office: In the interests of the quality of life of our entire community, we have created the opportunity to obtain free professional psychological help for students and university employees who feel the need for support.

e-mail: [POMOCPSYCHOLOGICZNA@SGH.WAW.PL](mailto:POMOCPSYCHOLOGICZNA@SGH.WAW.PL)

https://www.sgh.waw.pl/pomoc-psychologiczna

4. Cardinal Stefan Wyszyński University in Warsaw

PSYCHOLOGICAL SUPPORT: Every student who experiences difficulties in functioning in an academic environment can take advantage of free psychological advice and consultations provided by specialists from our University.

e-mail: [promocja@uksw.edu.pl](mailto:promocja@uksw.edu.pl) (Polish only)

<https://uksw.edu.pl/studenci/wsparcie-psychologiczne/>

5. Kozminski University (Business school in Warsaw)

Emergency Support of Personal Well-being: Provides free psychological and psychiatric assistance through the MindMed Institute. Students can access up to three free consultations with qualified specialists, either in-person or via video consultation. Follow-up sessions are chargeable

e-mail:

Marzena Indra: 600 328 934; [marzena.indra@kozminski.edu.pl](mailto:marzena.indra@kozminski.edu.pl)

Michał Godlewski: 538 510 074; [mgodlewski@kozminski.edu.pl](mailto:mgodlewski@kozminski.edu.pl)

https://www.kozminski.edu.pl/en/emergency-support-personal-well-being

6. Warsaw University of Life Sciences

Psychological consultations for students of SGGW: Psychological consultations are dedicated to all students at SGGW who want to solve psychological problems that are important to them, discover their causes and make positive changes in their lives.

Telephone registration 660 266 413 (No e-mail contact information)

<https://www.sggw.edu.pl/studenci/wsparcie-dla-studentow/pomoc-psychologiczna/>

7. Akademia WIT (Wyższa Szkoła Informatyki Stosowanej i Zarządzania w Warszawie)

Psychological support: WIT provides free psychological support for all University students.

e-mail: [B.Andrzejewska@wit.edu.pl](mailto:B.Andrzejewska@wit.edu.pl)

<https://www.wit.edu.pl/studenci/wsparcie-psychologiczne>

8. AWF Warsaw (AKADEMIA WYCHOWANIA FIZYCZNEGO JÓZEFA PIESUDSKIEGO W WARSZAWIE)

Psychological support: The main goal of the team's activity is to support students in selecting the appropriate professional form of therapy, which helps to mobilize their own and the University's resources in a situation of crisis and stress.

Pedagogical and Psychological Information Support Team

- Ph.D. Joanna Femiak [joanna.femiak@awf.edu.pl](mailto:joanna.femiak@awf.edu.pl)

- Dr. Katarzyna Płoszaj [katarzyna.ploszaj@awf.edu.pl](mailto:katarzyna.ploszaj@awf.edu.pl)

- Dr. Aleksandra Buchholz   [aleksandra.buchholz@awf.edu.pl](mailto:aleksandra.buchholz@awf.edu.pl)

<https://www.awf.edu.pl/uczelnia/bezpieczenstwo/wsparcie-psychologiczne>

9. Warsaw School of Management

Offer of psychological support: the University provides an offer of individual psychological support for students in need.

e-mail: [psycholog@wsz-sw.edu.pl](mailto:psycholog@wsz-sw.edu.pl)

<https://www.wsz-sw.edu.pl/glowna/wsparcie-psychologiczne/>

10. Akademia Techniczno-Artystyczna Nauk Stosowanych w Warszawie

Free psychological consultations for WSEIZ students

e-mail: [ewa.wisniewska@wseiz.pl](mailto:ewa.wisniewska@wseiz.pl)

<https://akademiata.pl/aktualnosci/bezplatne-konsultacje-psychologiczne-dla-studentow-wseiz/>

11. H. Chodkowska University of Technology and Commerce

Psychological consultations: for all students of the H. Chodkowska University of Technology and Commerce without leaving home.

e-mail: [przychodnia@terapiadialog.pl](mailto:przychodnia@terapiadialog.pl)

<https://www.uth.edu.pl/dla-studenta/biuro-ds-osob-niepelnosprawnych/konsultacje-psychologiczne>

12. Akademia Pedagogiki Specjalnej (Academy of Special Education named after Maria Grzegorzewska)

Psychological support for students from Ukraine

E-mail: [lzablocka@aps.edu.pl](mailto:lzablocka@aps.edu.pl)  and [mlukaszewska@aps.edu.pl](mailto:mlukaszewska@aps.edu.pl)

<https://www.aps.edu.pl/aktualnosci/wsparcie-psychologiczne-dla-studentek-studentow-z-ukrainy/>

13. Wojskowa Akademia Techniczna (Military Technical Academy)

Free psychological help for civilian students

TEL: phone: 261 839 918, mobile: 887 861 364

E-mail: [sekretariat.rektora@wat.edu.pl](mailto:sekretariat.rektora@wat.edu.pl) (Not specific to psychological support)

https://www.wojsko-polskie.pl/wat/articles/komunikaty-dla-studentow/bezplatna-pomoc-psychologiczna-dla-studentow-cywilnych/

14. UNIWERSYTET KARDYNALA STEFANA WYSZYNSKIEGO W WARSZAWIE (Cardinal Stefan Wyszyński University in Warsaw)

PSYCHOLOGICAL COUNSELING

E-mail: [bon@uksw.edu.pl](mailto:bon@uksw.edu.pl)

<https://bon.uksw.edu.pl/dla-studentow/poradnictwo-psychologiczne/>

15. Academy of Fine Arts in Warsaw

Psychological support, crisis assistance and helplines

E-mail: [Komisji.rownosc@asp.waw.pl](mailto:komisja.rownosc@asp.waw.pl)

<https://asp.waw.pl/studenckie-sos/>

16. Akademia Sztuki Wojennej (Academy of War Art)

Psychological support for students and employees of ASzWoj

E-mail: [b.drapikowska@akademia.mil.pl](mailto:b.drapikowska@akademia.mil.pl)

https://www.wojsko-polskie.pl/aszwoj/articles/aktualnosci-2/wsparcie-psychologiczne-dla-studentow-i-pracownikow-aszwoj/

Lubin

17. Uczelnia Jana Wyzykowskiego

Psychological support for UJW students: Students,

Our University offers you psychological support.

If you are in a mental crisis, need psychological support or help in adapting to new challenges, make an appointment with our expert, psychologist Dr. Renata Socha.

e-mail: [r.socha@ujw.pl](mailto:r.socha@ujw.pl)

<https://ujw.pl/wsparcie-psychologiczne-dla-studentow-ujw/>

18. POLITECHNIKA LUBELSKA (Lublin University of Technology) PSYCHOLOGICAL SUPPORT: WE REMIND YOU THAT PSYCHOLOGICAL SUPPORT IS FREE.

E-mail: [m.golawska@pollub.pl](mailto:m.golawska@pollub.pl)

<https://pollub.pl/studenci/organizacje/samorzad-studencki/wsparcie-psychologiczne>

19. KATOLICKI UNIWERSYTET LUBELSKI JANA PAWEA II (Catholic University of Lublin)

Psychological support: It is worth remembering that this support is completely free! It is provided by the Catholic University of Lublin Psychologist. (Not only for students, but for general public)

e-mail: psycholog@kul.pl; [sylwia.kedra@kul.pl](mailto:sylwia.kedra@kul.pl) https://www.kul.pl/wsparcie-psychologiczne-czym-jest-i-dlaczego-warto-z-niego-skorzystac,art_105028.html

20. UNIWERSYTET PRZYRODNICZY w Lublinie

free online consultations for Ukrainian students and doctoral students (from any university)

E-mail: pomoc-ukrainie@aps.edu.pl

https://up.lublin.pl/blog/wsparcie-psychologiczne-dla-ukrainskich-studentow/

Gdansk

21. Gdańsk University of Technology

Psychological Assistance Center: Every student and PhD student at the Gdańsk University of Technology can take advantage of free psychologist and psychotherapist's help.

Contact: [a.burkiewicz@gumed.edu.pl](mailto:a.burkiewicz@gumed.edu.pl)

https://pg.edu.pl/en/students/disability/psychological-support#:~:text=Students%20seeking%20psychological%20help%20are,free%20psychologist%20and%20psychotherapist's%20help.

22. University of Gdańsk

- Academic Psychological Support Center: As part of the ACWP UG activities, students, PhD students and employees can benefit from free psychological support

Contact: [cwp@ug.edu.pl](mailto:cwp@ug.edu.pl)

<https://centrumwsparcia.ug.edu.pl/o-nas/>

Poznan

23. Adam Mickiewicz University (AMU)

- AMU Psychological Development and Support

AMU Psychological Development and Support offers a wide range of

assistance in dealing with challenging situations in life on different levels through adaptation to a new university environment, relationships with others, and personal and family concerns.

MAIL US: [poradniariwp@amu.edu.pl](mailto:poradniariwp@amu.edu.pl)

https://amu.edu.pl/en/education/psychological-assistance/psychological-development-and-support

24. Poznań University of Life Sciences

-Support and Development Center: Personal development, career counselling, developing cooperation with employers, broadly understood psychological support, including support in difficult and/or crisis situations that violate a person's sense of security, improving the accessibility of the University for people with disabilities - these are just examples of support that can be used by students, university staff and graduates of the Poznań University of Life Sciences, referred to in Order No. 14/2020 of the Rector of the Poznań University of Life Sciences of 3 February 2020 establishing the Support and Development Centre (CWR). (Translation from Polish)

Contact: [cwr@up.poznan.pl](mailto:cwr@up.poznan.pl)

<https://puls.edu.pl/page/centrum-wsparcia-i-rozwoju>

25. Faculty of Psychology and Cognitive Science

Development and Mental Support Clinic for UAM students: The clinic offers short- and medium-term psychological and therapeutic assistance for students, PhD students and employees of Adam Mickiewicz University in Poznań.

e-mail: poradniariwp@amu.edu.pl

<https://psychologia.amu.edu.pl/dla-studenta/wsparcie/pomoc-psychologiczna-na-uam>

26. Academy of Physical Education in Poznań

The Office for Supporting People with Disabilities: Psychological support for students, PhD students and AWF employees

The Office for Supporting People with Disabilities offers all students, doctoral students and employees of the AWF the opportunity to use professional psychological help free of charge.

e-mail: [office@awf.poznan.pl](mailto:office@awf.poznan.pl)

https://awf.poznan.pl/wsparcie-psychologiczne-dla-studentow-doktorantow-i-pracownikow-awf/

27. WSB Merito University in Poznań (private University for finance, banking, economics, and entrepreneurship)

Psychological Support WSB: Psychologists from WSB offer support and psychological consultations for students and lecturers who, due to the pandemic and social quarantine, are going through difficult times, experiencing loneliness, anxiety or emotional crisis.

Email(counseling specialist contracts, there are 6 more):

Patrycja Walenczak: patrycja.walenczak@wsb.poznan.pl

Anna Suchariska: anna.suchanska@wsb.poznan.pl

Iwona Werner: iwona.werner@wsb.poznan.pl

https://www.merito.pl/poznan/aktualnosci/wsparcie-psychologiczne-wsb

28. Poznań University of Economics

Psychological Consultant for the Study Process

 e-mail: [julita.wojciechowska@amu.edu.pl](mailto:julita.wojciechowska@amu.edu.pl)

https://ue.poznan.pl/studenci/psychologiczny-konsultant-ds-procesu-studiowania/

29. University of Arts in Poznań

Psychological consultations for students and students: All students of the Magdalena Abakanowicz University of Arts in Poznań can benefit from free psychological consultations.

e-mail: [anna.olszewska-konopa@uap.edu.pl](mailto:anna.olszewska-konopa@uap.edu.pl)

<https://uap.edu.pl/dla-studentow/konsultacje-psychologiczne-dla-studentow/>

30. WYZSZA SZKOLA BEZPIECZENSTWA (HIGHER SCHOOL OF SECURITY) in Poznan

Psychological support: A student with a disability certificate has the opportunity to benefit from psychological support provided by the Higher School of Security.

e-mail: [niepelnosprawni@wsb.net.pl](mailto:niepelnosprawni@wsb.net.pl)

https://www.wsb.net.pl/studenci-z-niepelnosprawnoscia/wsparcie-psychologiczne/

Torun

31. UNIWERSYTET MIKOLAJA KOPERNIKA W TORUNIU

UNIVERSITY CENTER FOR SUPPORT AND PERSONAL DEVELOPMENT: Free psychological consultations (on-site or online) for students at UMK and PhD students

e-mail: e-mail:  [osrodek_wspiera@umk.pl](mailto:osrodek_wsparcia@umk.pl)

https://wsparcie.umk.pl/pages/pomoc_psychologiczna/

Bydgoszcz

32. POLITECHNIKA BYDGOSKA im. Jana i Jedrzeja Sniadeckich

Psychological help: Mental health is just as important as physical health - that's why, guided by the well-being of our students, we inform about the possibility of free consultation meetings with a psychologist.

Marta Krakowiak, M.A., e-mail: [psycholog@pbs.edu.pl](mailto:psycholog@pbs.edu.pl)

https://pbs.edu.pl/pl/student/sprawy-studenckie/pomoc-psychologiczna

33. Kazimierz Wielki University in Bydgoszcz

Rector's Plenipotentiary for Psychological Assistance: Students and employees of Kazimierz Wielki University in Bydgoszcz can use psychological assistance free of charge.

Dr. Kamilla Komorowska

e-mail: kamkom@ukw.edu.pl

<https://www.ukw.edu.pl/strona/aktualnosci/archiwum/64740/pomoc-psychologiczna-dla-studentow-i-pracownikow-ukw>

Gliwice

34. Silesian University of Technology in Gliwice

Psychological consultations at the Silesian University of Technology: All students and PhD students of the Silesian University of Technology can take advantage of free psychological consultations (in stationary or remote).

e-mail: [bon@polsl.pl](mailto:bon@polsl.pl)

<https://www.polsl.pl/rd1-cos/bonkonpsych/>

Katowice

35. University of Silesia in Katowice

Faculty of Social Sciences: Students and PhD students of the University of Silesia can use free psychological counseling and advice at the Student Service Center and the Careers Office

e-mail: [psycholog@us.edu.pl](mailto:psycholog@us.edu.pl)

<https://us.edu.pl/wydzial/wns/pomoc-psychologiczna/>

Szczecin

36. WSB Merito Szczecin University

Psychological consultations for students

e-mail: [pomagamy@centrumunitas.pl](mailto:pomagamy@centrumunitas.pl)

<https://www.merito.pl/szczecin/aktualnosci/konsultacje-psychologiczne-dla-studentow>

37. Zachodniopomorski Uniwersytet Technologiczny w Szczecinie (West Pomeranian University of Technology in Szczecin)

Psychological help for students

e-mail: [bon@zut.edu.pl](mailto:bon@zut.edu.pl)

https://www.pm.szczecin.pl/pl/studenci/akademickie-centrum-wsparcia/

Zielona Góra

38. University of Zielona Góra > Faculty of Social Sciences

Students for Students: Help and Psychological Support: The offer is addressed to all students, regardless of their field of study, specialization, interests, gender or views. Fifth-year psychology students, under the substantive supervision of experienced psychologists, will provide professional support:

e-mail: [pomoc_psych@wns.uz.zgora.pl](mailto:pomoc_psych@wns.uz.zgora.pl)

https://wns.uz.zgora.pl/aktualnosci/studenci-studentom--pomoc-i-wsparcie-psychologiczne-229.html

Łódź

39. UNIWERSYTET LÓDZKI (University of Lod)

SUPPORT AND ACCESSIBILITY CENTRE: Provides free psychological help for individuals facing difficult life situations. The centre can be contacted directly for assistance

[psychological support at the ACW UL](https://www.uni.lodz.pl/fileadmin/Jednostki/ACW/ZASADY_WSPARCIA_PSYCHOLOGICZNEGO_EN.pdf).

email: [cwid@uni.lodz.pl](mailto:cwid@uni.lodz.pl)

https://www.uni.lodz.pl/en/strefa-doktoranta/wsparcie/academic-support-centre

40. WYZSZA SZKOKA Biznesu i Nauk o Zdrowiu w todzi (College of Business and Health Sciences)

Free psychological support

e-mail is not shared but application form is available (in the link)

<https://www.wsbinoz.edu.pl/dla-studenta/bezplatne-wsparcie-psychologiczne>

41. Politechnika kódzka

Psychological support

Signed up form not working

https://bon.p.lodz.pl/wsparcie/wsparcie-psychologiczne

Wrocław

42. University of Wrocław

Psychological Counselling Centre: Students and PhD students of the University of Wrocław will also be able to count on free psychological support in the Psychological Counselling Centre run by associate professor Alina Czapiga.

e-mail: [poradnia.psychologiczna@uwr.edu.pl](mailto:poradnia.psychologiczna@uwr.edu.pl)

https://uwr.edu.pl/poradnia-psychologiczna-dla-studentow-i-doktorantow-znow-dziala/

43.Politechnika Wrocławska (Science and Technology)

Psychological clinic for students of Wrocław University of Science and Technology

e-mail: [pomoc.n@pwr.edu.pl](mailto:pomoc.n@pwr.edu.pl)  (not specific to psycholocaical clinic)

<https://ddo.pwr.edu.pl/dla-studentow/wsparcie-psychologiczne>

44. [University of Lower Silesia DSW Wrocław](https://www.dsw.edu.pl/)

Support, Psychological Help and Psychoeducation Center: Anyone with an active student status at the University can take advantage of the intervention support program. Those on dean's leave can also take advantage of the support.

e-mail: [centrumwspiera@dsw.edu.pl](mailto:centrumwsparcia@dsw.edu.pl)

<https://www.dsw.edu.pl/strefa-studenta/centrum-wsparcia-pomocy-psychologicznej-i-psychoedukacji>

45. UNIWERSYTET PRZYRODNICZY WE WROCEAWIU (University of Environmental and Life Sciences in Wrocław)

Psychological help

E-mail: [psycholog@upwr.edu.pl](mailto:psycholog@upwr.edu.pl)

https://upwr.edu.pl/studia/wsparcie-dla-studenta/biuro-rownosci-i-wsparcia/pomoc-psychologiczna

46. AKADEMIA MUZYCZNA im. Karola Lipinskiego we Wroctawiu (Karol Lipiński Academy of Music in Wrocław)

Psychological support for students from Ukraine

E-mail: [pomoc-ukrainie@aps.edu.pl](mailto:pomoc-ukrainie@aps.edu.pl)

<https://amuz.wroc.pl/wsparcie-psychologiczne-dla-studentow-ukrainy-7419>

47. Uniwersytet Ekonomiczny we Wroctawiu ([Wrocław University of Economics](https://www.ue.wroc.pl/copyright.html))

Psychological consultations for students, graduates and EU employees

e-mail: [biurokarier@ue.wroc.pl](mailto:biurokarier@ue.wroc.pl)

<https://www.ue.wroc.pl/aktualnosci/8164/konsultacje_psychologiczne_dla_studentow_absolwentow_i_pracownikow_ue.html>

48. AKADEMIA WYCHOWANIA FIZYCZNEGO IM. POLSKICH OLIMPIJCZYKÓW WE WROCLAWIU (Academy of Physical Education named after Polish Olympians)

Psychological support for students and employees

TEL: 512743604 (no e-mail contact)

<https://awf.wroc.pl/pomoc-psychologiczna-dla-studentow-i-pracownikow/>

49. Uniwersytet Dolnoslaski DSW Wroctaw (University of Lower Silesia DSW)

Psychological support: The Center provides free psychological counseling, crisis intervention and psychoeducation.

No-contact available. Registration form in the link

<https://www.dsw.edu.pl/strefa-studenta/wsparcie-osob-z-niepelnosprawnoscia/zakres-wsparcia>

50. UNIWERSYTET PRZYRODNICZY WE WROCEAWIU (University of Environmental and Life Sciences in Wrocław)

Free psychological consultations for students

<https://thepresja.pl/studenci-dla-zdrowia/> (registration site)

https://upwr.edu.pl/ogloszenia/bezplatne-konsultacje-psychologiczne-dla-studentow-listopad-2024-727.html

Krakow

51. Jagiellonian University in Krakow

Student support and adaptation center: Support at the center is free of charge and intended for all students at Jagiellonian University.

Email: [sowa@uj.edu.pl](mailto:sowa@uj.edu.pl)

<https://bezpieczni.uj.edu.pl/sytuacje-kryzysowe>

52. University of the National Commission in Krakow

students and PhD students of the University of the National Commission in Krakow can take advantage of free, ad hoc psychological consultations.

e-mail: [bon@uken.krakow.pl](mailto:bon@uken.krakow.pl)

<https://www.uken.krakow.pl/student/aktualnosci-dla-studentow/6068-wsparcie-psychologiczne-dla-studentow-i-doktorantow-uken>

53. Akademia Sztuk Pieknych im. Jana Matejki w Krakowie (The Academy of Fine Arts in Kraków) Psychological support

E-mail: [pomocpsychologiczna@asp.krakow.pl.](mailto:pomocpsychologiczna@asp.krakow.pl.)

<https://www.asp.krakow.pl/wsparcie-psychologiczne/>

54. Politechniki Krakowskiej (CRACOW UNIVERSITY OF TECHNOLOGY)

Psychological support (may not be in practice anymore. The detail page is unavailable.)

E-mail: [bon@pk.edu.pl](mailto:bon@pk.edu.pl) (Not specific to psychological support)

<https://bon.pk.edu.pl/studenci/wsparcie-psychologiczne/>

55. Akademia Kultury Fizycznej im. Bronistawa Czecha w Krakowie (Academy of Physical Culture)

Psychological assistance at AWF

E-mail: [marta.kuleta@awf.krakow.pl](mailto:marta.kuleta@awf.krakow.pl)

<https://www.awf.krakow.pl/dla-studentow/pomoc-psychologiczna>

56. KRAKOWSKI OSRODEK DORADZTWA DLA ARTYSTÓW (Krakow Academy of Music)

Psychological support for students of the Medical Academy in Krakow

E-mail:[koda@koda.krakow.pl](mailto:koda@koda.krakow.pl)

<https://koda.krakow.pl/page/wsparcie_psychologiczne_dla_studentow_am_w_krakowie>

57. Akademia Sztuk Teatralnych im. St. Wyspianskiego w Krakowie

PSYCHOLOGICAL CONSULTATIONS FOR STUDENTS

E-mail: [psycholog@ast.wroc.pl](mailto:psycholog@ast.wroc.pl)

<https://www.ast.wroc.pl/studenci/wsparcie-psychologiczne/>

58. Dzial Dostepnosci AGH (AGH University of Science and Technology Accessibility Department)

Psychological support: Support for students with psychological difficulties

E-mail: [anna.lulek@agh.edu.pl](mailto:anna.lulek@agh.edu.pl)

<https://bon.agh.edu.pl/p,110,wsparcie-psychologiczne>

59. [John Paul II Pontifical University in Krakow](https://bon.upjp2.edu.pl/)

Psychological support

The Office for People with Disabilities offers psychological support to students and doctoral students.

E-mail: [psycholog@upjp2.edu.pl](mailto:psycholog@upjp2.edu.pl)

<https://bon.upjp2.edu.pl/studenci--doktoranci---wsparcie/wsparcie-psychologiczne>

60. UNIWERSYTET ROLNICZY im. Hugona Koltataja w Krakowie (Hugo Kołłątaj University of Agriculture in Krakow)

Psychological help for students

E-mail: [kinga.zych@urk.edu.pl](mailto:kinga.zych@urk.edu.pl) or [wiktor.potoczny@urk.edu.pl](mailto:wiktor.potoczny@urk.edu.pl)

<https://urk.edu.pl/student/pomoc-psychologiczna-dla-studentow>k

Częstochowa

61. Częstochowa University of Technology

Psychological support: The Rector's Representative for Psychological Support – Dr. Małgorzata Randak – Jezierska informs students, doctoral students and employees of the Częstochowa University of Technology about the possibility of using free psychological consultations.

e-mail: [psycholog@pcz.pl](mailto:psycholog@pcz.pl)

https://wis.pcz.pl/student/wsparcie-studenta/wsparcie-psychologiczne

Rzeszów

62. University of Rzeszów

Psychological support of the No Stress Psychological Counseling Center: The detail page is "Error 404.", Maybe it was discontinued.

e-mail : [dochojska@ur.edu.pl](mailto:dochojska@ur.edu.pl)

<https://www.ur.edu.pl/pl/student/strefa-wsparcia>

Wałbrzych

63. University of Management and Entrepreneurship in Wałbrzych

Support for students – PSYCHOLOGICAL SUPPORT: The Wałbrzych-based Higher School of Management and Entrepreneurship implements many activities aimed at supporting students and the academic community in everyday life, including in situations of crisis, trauma, mourning, sudden change in life or illness.

e-mail: [psychologist@wwszip.pl](mailto:psycholog@wwszip.pl)

<https://wwszip.pl/wsparcie-dla-studentow-wsparcie-psychologiczne/>

Piła

64. Akademia Nauk Stosowanych im. Stanistawa Staszica w PileNon-university (Academy of Applied Sciences

named after Stanisław Staszic in Piła)

Launch of Psychological Support for Students

E-mail: [dlupicka@ans.pila.pl](mailto:dlupicka@ans.pila.pl) (Dr. Dagmara Łupicka-Szczęśnik)

<https://bon.ans.pila.pl/studenci-z-niepelnosprawnosciami/wsparcie-psychologiczne-dla-studentow-ans-w-pile>

Siedlce

65. Uniwersytet w Siedlcach (University of Siedlce)

Psychological support: We offer our students individual psychological consultations.

TEL: 782 354 844 (to make an appointment)

E-mail: [cwn@uph.edu.pl](mailto:web@support.com) (Not specific to psychological support)

<https://cwn.uph.edu.pl/psycholog>

Kielce

66. Wydziat Budownictwa i Architektury (Kielce University of Technology)

> Faculty of Construction and Architecture

Free psychological consultations for students

E-mail: [wbia@tu.kielce.pl](mailto:wbia@tu.kielce.pl) (Not specific to psychological support)

<https://wbia.tu.kielce.pl/bezplatne-konsultacje-psychologiczne-dla-studentow/>

67. The University of Public Administration in Kielce

Psychological support for students and employees

E-mail: [psycholog@wsap-kielce.edu.pl](mailto:psycholog@wsap-kielce.edu.pl)

<https://wsap-kielce.edu.pl/wsparcie-psychologiczne-dla-studentow-i-pracownikow/>

Słupsk

68. Pomeranian University in Słupsk and the Maria Grzegorzewska Academy of Special Education in Warsaw

Free psychological support for students from Ukraine

E-mail: [pomoc-ukrainie@aps.edu.pl](mailto:pomoc-ukrainie@aps.edu.pl)

<https://www.upsl.edu.pl/ukraina/pomoc-psychologiczna-psihologichna-dopomoga/bezplatne-wsparcie-psychologiczne-dla-studentek-i-studentow-z-ukrainy-bezkoshtovna-psihologichna-pidtrimka-dlya-studentok-ta-studentiv-z-ukrayini>

Białystok

69. University of Bialystok > Faculty of Physics

Psychological and mental support for students

E-mail: [sklep@uwb.edu.pl](mailto:fizyka@uwb.edu.pl) (Not specific to psychological support)

<https://fizyka.uwb.edu.pl/studenci/dokumenty-informacje-wydzialowe-dla-studentow/wsparcie-psychologiczne-i-psychiczne-studentow>

70. BIALYSTOK UNIVERSITY OF TECHNOLOGY

Free psychological consultations for students: The registration site doesn't work.

E-mail: [rectorate@pb.edu.pl](https://pb.edu.pl/kontakt/dane-teleadresowe/)

<https://pb.edu.pl/polecamy/bezplatne-konsultacje-psychologiczne-dla-studentow/>

Tarnów

71. Tarnów Academy

ONLINE PSYCHOLOGICAL HELP FOR STUDENTS

E-mail: [serwispsychologiczne@psrp.org.pl](mailto:wsparciepsychologiczne@psrp.org.pl)

<https://anstar.edu.pl/pomoc-psychologiczna-online-dla-studentow/>

Biała Podlaska

72. AKADEMIA BIALSKA NAUK STOSOWANYCH IM.JANA PAWEA II

Due to the spread of the SARS-CoV-2 virus,

we have prepared a list of specialist clinics to meet the mental health needs and psychological support of students and university employees.

TEL: 83 344 51 50 (clinic number)

<http://www.pswbp.pl/index.php/aktualnoci-prac/71-aktualnoci-pracownicy/10559-wsparcie-psychologiczne-dla-studentow-i-pracownikow-uczelni>

Łomża

73. WYDZIAL NAUK SPOLECZNYCH I HUMANISTYCZNYCH AKADEMII LOMZYNSKIEI (University of Lomza)

Psychological help: Free psychological help provided by psychologists associated with the University can be obtained through the Academic Center for Personal and Social Development

E-mail: [focus@al.edu.pl](mailto:focus@al.edu.pl)

https://al.edu.pl/wnsh/student/pomoc-psychologiczna

Płock

74. Warsaw University of Technology, Płock Branch

Psychological support: Warsaw University of Technology offers free psychological support for all students, PhD students and university employees

E-mail: [wspiera.sp@pw.edu.pl](mailto:wsparcie.sp@pw.edu.pl)

<https://www.pw.plock.pl/Studenci/Sekretariat-KNEiS/Wsparcie-psychologiczne>

Opole

75. POLITECHNIKA OPOLSKA ([Opole University of Technology](http://po.opole.pl/))

[Psychological help for all students of the Opole University of Technology](https://wsparcie.po.opole.pl/index.php/aktualnosci/58-pomoc-psychologiczna-dla-studentow-z-ukrainy)

E-mail: wspiera@po.edu.pl

<https://wsparcie.po.opole.pl/>

Gdni

76. WYZSZA SZKOLA KOMUNIKACJI SPOLECZNEJ W GDYNI

Free psychological consultations for students

<https://wsks.pl/bezplatne-konsultacje-psychologiczne-dla-studentow/>

Jarosław

77. Państwowa Akademia Nauk Stosowanych im. ks. Bronisława Markiewicza w Jarosławiu (The State Academy of Applied Sciences named after Rev. Bronisław Markiewicz in Jarosław)

Free psychological support for students and employees

TEL:16 624 96 40

E-mail: [university@pansjar.edu.pl](mailto:uczelnia@pansjar.edu.pl) (Not specific to psychological support)

<https://www.pwste.edu.pl/2022/01/14/bezplatne-wsparcie-psychologa-dla-studentow-i-pracownikow/>

Czestochowie

78. Uniwersytet Jana Dtugosza w Czestochowie > Uczelniana Rada Samorzadu Studenckiego

Free psychological consultations for students: *detail page doesn’t work. Contact not provided.

<http://www.urss.ujd.edu.pl/n1035,Bezplatne-konsultacje-psychologiczne-dla-studentow>

Nysa

79. PAÑSTWOWA AKADEMIA NAUK STOSOWANYCH W NYSIE (the State Academy of Applied Sciences in Nysa)

Psychological support for students

E-mail: [grzegorz.kulik@pans.nysa.pl](mailto:grzegorz.kulik@pans.nysa.pl) (Mr. Grzegorz Kulik)

<https://pans.nysa.pl/org/stypendia/strona/wsparcie-psychologiczne-dla-studentow>

Koszalin

80. Koszalin University of Technology > Faculty of Electronics and Computer Science

Academic Psychological Support for Students of the Koszalin University of Technology

E-mail:  [akademickapomoc@tu.koszalin.pl](mailto:agnieszka.rydzkowska@tu.koszalin.pl)

<https://tu.koszalin.pl/weii/kat/1193/akademickie-wsparcie-psychologiczne>

Oświęcimiu

81. Matopolska Uczelnia Panstwowa im. rtm. Witolda Pileckiego w Oswiecimiu (Malopolska State University named after Capt. Witold Pilecki)

Psychological support

E-mail: (Ms. Maja Piekut) [maja.piekut@mup.edu.pl](mailto:maja.piekut@mup.edu.pl)

https://uczelniaoswiecim.edu.pl/studia/wsparcie-studentow/wsparcie-psychologiczne/

Students' Parliament of the Republic of Poland

*Many universities refer students to this website.

-"PSRP Comfort Zone" : Online psychological help for students from all over Poland – this is a new initiative of the Students' Parliament of the Republic of Poland, which has prepared the "PSRP Comfort Zone" campaign. Every student can use online therapy and take part in webinars led by specialists. The project was co-financed by the Ministry of Science and Higher Education.

Contact:  [serwispsychologiczne@psrp.org.pl](mailto:wsparciepsychologiczne@psrp.org.pl)

https://wsparciepsychologiczne.psrp.org.pl/
